# Measuring the psychosocial, biological, and cognitive signatures of profound stress in humanitarian settings: impacts, challenges, and strategies in the field

**DOI:** 10.1186/s13031-020-00286-w

**Published:** 2020-06-23

**Authors:** Catherine Panter-Brick, Mark Eggerman, Alastair Ager, Kristin Hadfield, Rana Dajani

**Affiliations:** 1 Department of Anthropology & Jackson Institute of Global Affairs, Yale University, New Haven, USA; 2 MacMillan Center for International and Area Studies, Yale University, New Haven, USA; 3 Institute for Global Health and Development, Queen Margaret University, Edinburgh, UK; 4 Department of Biological and Experimental Psychology, Queen Mary University of London, London, UK; 5 Department of Biology and Biotechnology, Hashemite University, Zarqa, Jordan

**Keywords:** Adolescent, Conflict, Cognition, Cortisol, Impact evaluation, Longitudinal, Mental health, Partnership, Refugee, Resilience, Stress, War

## Abstract

**Background:**

Evidence of ‘what works’ in humanitarian programming is important for addressing the disruptive consequences of conflict and forced displacement. However, collecting robust scientific evidence, and ensuring contextual relevance, is challenging. We measured the biological, psychosocial, and cognitive impacts of a structured psychosocial intervention, implemented by Mercy Corps with Syrian refugees and Jordanian host-community youth. In this paper, we present a case analysis of this evaluation study and reflect on the scientific contributions of the work, the challenges experienced in its delivery, and the strategies deployed to address them.

**Discussion:**

We identified challenges with respect to study design, methods, and dissemination: these included the logistics and acceptability of implementing a randomized controlled trial in a humanitarian context, the selection and refinement of culturally-relevant research tools and community-based practices, and the dissemination of results to multiple stakeholders. We demonstrated beneficial and sustained impacts on self-reports of insecurity, stress, and mental health; developed a reliable and culturally-relevant measure of resilience; experimentally tested cognitive skills; and showed that levels of cortisol, a biomarker of chronic stress, reduced by one third in response to intervention. Using stress biomarkers offered proof-of-concept evidence, beyond self-reported data: interventions targeting mental health and psychosocial wellbeing can regulate physiological stress in the body as well as improve self-reported mental health and wellbeing. We built constructive dialogue between local communities, scholars, humanitarian practitioners, and policy-makers.

**Conclusions:**

Our work shows the value of rigorous research in humanitarian settings, emphasizing relevance for local communities and meaningful ways to build research ownership. Findings encourage the adoption of cognitive measures and stress biomarkers alongside self-report surveys in evaluating programme impacts. High-quality scientific research with youth can be feasible, useful, and ethical in humanitarian settings.

## Background

The process of collecting robust scientific evidence of ‘what works’ in humanitarian programming, and of ensuring contextual relevance in local communities, can be very challenging. In this paper, we present a case analysis of a longitudinal, interdisciplinary research project conducted with Syrian refugee and Jordanian non-refugee youth living in urban communities near active war zones. Responding to calls to strengthen the evidence base for public health interventions in humanitarian settings, [1, 2] our research team worked at the invitation of Mercy Corps to evaluate one of its youth-focused humanitarian programmes - a brief, scalable, community-based intervention aimed at improving the mental health and psychosocial wellbeing of war-affected adolescents. Subsequent to the completion of a randomized controlled trial, the team engaged in a systematic reflection on the research experience. We reviewed the scientific contributions of the study, the challenges in its implementation, and the strategies adopted to address them.

### Humanitarian context and programme response

Worldwide, there are now 70.8 million forcibly displaced people, over half of whom are children under 18 years of age [3]. The Syrian crisis has created the world’s largest refugee crisis since World War II, with over 5.5 million people forced to flee their country since 2011 [4]. To-date, 656,700 Syrians are taking refuge in neighboring Jordan, 81% of whom live as urban refugees outside demarcated camps [5]. For children and youth, conflicts such as the Syrian crisis present urgent challenges for survival, health, and development pathways [6] – an entire generation of young Syrians is deemed at-risk for developmental and health consequences of exposure to trauma and toxic stress [7]. In 2013, the *No Lost Generation* initiative, a vigorous humanitarian response led by UNICEF, Mercy Corps, Save the Children, and World Vision, launched a number of youth-focused, community-based interventions to reach war-affected adolescents in crisis settings in the Middle East region [8].

As part of this initiative, Mercy Corps implemented the *Advancing Adolescents* programme with conflict-affected youth in Jordan, Lebanon, Iraq, Syria, and Turkey. This psychosocial intervention offers structured activities (16 sessions, across 8 weeks) to both Syrian refugee and host-community youth, with the goal of alleviating profound stress, enhancing psychosocial support, strengthening learning skills, and building resilience and social cohesion [9]. The program draws on a profound stress attunement framework to promote capacities for mediating extreme and prolonged stress, following the International Child Development Programme developed in Holland for global use [10]. Key elements of the program are common to other psychosocial interventions, including group-based sessions (developing technical, vocational, crafts, or fitness skills) run by trained, lay community workers. The latter were Jordanian and Syrian men and women, aged 21+, recruited from local community-based NGOs; prior to implementation, they completed the 16-day Coaches Foundation Training Programme delivered by Mercy Corps. The Mercy Corps training manual covers practicing healthy communication; defining profound stress, its impact on the human brain, and principles of attunement; developing gender equity and adolescent protection; building psychosocial resilience; and enhancing skills of creative facilitation for effective technical training [10].

### Research study

Our study aimed to (a) test impacts of *Advancing Adolescents* on mental health and psychosocial wellbeing, and (b) develop a useful and sensitive toolkit for interdisciplinary evaluation. This would address important questions for humanitarian practice and policy (*Does the intervention work, for whom, and for how long? What are the best ways to generate robust evidence of what works in humanitarian settings?)*. Before this, programme impact evaluations in the region had been wholly based on the self-reports of beneficiaries - sole reliance on self-reports can lead to response bias, especially where programme implementations and evaluations are done by the same agency, and sole reliance on beneficiaries, in the absence of a control group, can lead to unreliable evidence of impacts. In response to the need for an innovative research design to track stress alleviation and learning skills for beneficiaries and non-beneficiaries, we used an interdisciplinary approach embedded in a randomized controlled trial: we measured how *Advancing Adolescents* impacted stress in the mind, the body, and the brain (in terms of felt experiences, physiological regulation, and cognitive function), for both refugee and non-refugee adolescents.

We formed a research consortium after consulting with local community leaders and obtaining formal approval from the prime minister’s office to work in northern Jordan, drawing together international and regional staff at Mercy Corps; scholars from the UK, USA, and Canada; local community-based organizations running youth centers; and an independent NGO (Taghyeer, or We Love Reading) with research and advocacy experience in disadvantaged communities. Taghyeer anchored the project locally, by engaging communities in research design and implementation, and by recruiting a core research team of six Syrian and Jordanian women with university and professional degrees. We conducted our 18-month project (2015–17) in four northern cities (Irbid, Jarash, Mafraq, and Zarqa), receiving a significant influx of Syrian refugees. We implemented a quasi-experimental trial and then a randomized controlled trial with a waitlist control condition. We collected data at three time-points: baseline (T1), 10-weeks post-intervention (T2), and after 11 months (T3). We secured a gender-balanced sample of refugee and non-refugee youth (*n* = 817, 12–18 years) at T1, representing 20% of targeted beneficiaries and 48% of youth enrolled in *Advancing Adolescents* in Jordan at the time of study. With high levels of relocation in the targeted population, we retained 533 youth at T2 and 302 youth at T3. Our findings and methodologies, published in journals within the fields of biological sciences, child psychology and psychiatry, and child development, are summarized in Table 1.

**Table 1:** Summary of the research question, outcomes of interest, and findings for interdisciplinary evaluation of program impacts in a humanitarian setting

*Research question:* What are the psychosocial, biological, and cognitive impacts of a brief, scalable intervention for war-affected youth?	
*Mental health, insecurity, and psychosocial stress:* We used regionally and internationally relevant instruments such the Arab Youth Mental Health scale, Strength and Difficulties Questionnaire, Human Insecurity scale, Human Distress scale, and Perceived Stress Scale, in combination with a Trauma Events Checklist and Household Wealth Index. Youth who engaged in the *Advancing Adolescents* program reported fewer symptoms of psychosocial stress, insecurity, and mental health difficulties, relative to waitlisted controls in the randomized controlled trial [11]. We found small to moderate effect sizes across outcomes - equivalent or greater than intervention impacts reported for psychosocial interventions in this age-group in crisis settings [11]. We noted differential impacts for youth with high/low trauma exposures, and there were sustained effects over 11 months.	
*Traumatic stress and resilience:* We used the Child Revised Impact of Events Scale, and developed the Arabic-language Child and Youth Resilience Measure [12]. We found no program impacts for these outcomes, but resilience scores did moderate changes in anxiety/depression for youth with high trauma exposure [11].	
*Biological stress:* We used biomarkers such as hair cortisol secretion and immune function biomarkers to track chronic physiological stress. We found that average cortisol levels dropped by a third (38%) in response to the program; stress was down-regulated in youth with high cortisol levels and up-regulated in youth with blunted cortisol profiles, showing beneficial regularization over time [13, 14].	
*Cognitive function:* We used Rapid Assessment Cognitive and Emotional Regulation [RACER] tests, developed for use with children in low- and middle-income countries, [15] to measure working memory and inhibitory control, both important for learning abilities. We found no program impacts on these executive function skills, but showed that deficits in working memory were associated with pervasive poverty, rather than trauma exposure [16].	

## Discussion

### Scientific importance

Our study was the first to go beyond self-reports in measuring the effectiveness of a brief, scalable community-based program seeking to improve the psychosocial wellbeing of war-affected adolescents. Notably, it included biomarkers to track the cumulative effects of stress in the body: measuring cortisol concentrations in ~ 100 strands of hair, cut close to the scalp, reveals stress physiology in hair growth over the past month, with repeated sampling over time yielding cortisol secretion data akin to a stress diary. This helped us understand the processes of recovery from past trauma and current insecurity, showing that even brief interventions to improve day-to-day environments can protect adolescent health and wellbeing. Stress biomarkers were relatively simple and objective ways of helping to measure changes over time. They conclusively demonstrated that the Mercy Corps program had beneficial impacts for adolescents – not only did it improve self-reports of insecurity, stress, and poor mental health, but it changed and regulated physiological stress in the body.

In addition, our study tested cognitive skills and measured resilience – also a first for Syrian refugee youth. We found no evidence of program impacts for either outcome. This was an important lesson: programs that demonstrably alleviate profound stress, but not poverty per se, do not necessarily boost cognitive functioning, while programs that target individual-level changes may not change family and community environments enough to move the needle for psychosocial resilience. In order to boost learning skills and resilience in settings of protracted crises, humanitarian programs may need to broaden the package of youth-focused interventions with sustained support in home and school environments. We see that humanitarian programs do good in crisis settings, but often achieve only limited good given the multiple challenges of operating in such contexts. For this reason, evidence of impact or non-impact truly matters, helping to calibrate expectations and to move towards a more evidence-based model of humanitarian practice.

The field of mental health and psychosocial support (MHPSS) is poised to consolidate robust evidence on what works in humanitarian settings; specifically, it must assess why program impacts (often measured using self, parent, or teacher reports) are mostly small, variable, or nil, especially over the long-term [17]. Psychosocial interventions for children and adolescents are one of the most frequently implemented, yet poorly evaluated, interventions in crisis settings [1, 18, 19]. This proves frustrating for humanitarians looking for credible, actionable evidence in MHPSS programming. Our study introduced innovative, practical ways to generate robust scientific evidence and it also created strong partnerships between researchers, practitioners, and local communities. Syrian and Jordanian research team members were trained in research methods and analysis, engaged fully and regularly with US and UK partners to share cultural insights and benefit from opportunities created by international, collaborative exchange. We adopted a study design that prioritized both scientific rigor and local community engagement, used interdisciplinary methods of assessment to track multisystemic signatures of profound stress alleviation, and engaged in wide-ranging dissemination activities to promote the awareness of results and uptake of recommendations [20].

### Challenges

We faced challenges with respect to study design, methods, and dissemination (Table 2).

**Table 2:** Ethical, logistical, and temporal challenges of research in a humanitarian setting

*Study design:* Implementing a fair and transparent randomization process, given short and unpredictable funding timelines; obtaining reliable data to assess sample representativeness; sampling and tracking mobile refugee communities over the period of a year.	
*Methods:* Collecting biological samples and testing cognitive skills, without undue burden, in difficult field conditions; implementing a demanding interdisciplinary survey protocol, with a range of reliable and culturally-relevant psychometric scales, and introducing multiple biomarkers and cognitive testing to assess logistical feasility and cultural acceptability in humanitarian settings.	
*Dissemination:* Reconciling the different demands and time-frames, for dissemination of results in peer-reviewed academic journals vs. influential policy reports.	

#### Study design

A first set of challenges involved the ethical implementation of the randomized controlled trial and the logistics involved in sampling representative populations groups and tracking a mobile refugee population. Ethically, we could not randomly allocate participants to intervention or control groups, unless the latter were wait-listed to another cycle of program implementation. While *Advancing Adolescents* was implemented on a rolling basis, the Mercy Corps regional office was nearing the end of a funding cycle, unsure of its ability to extend programming for another year. Concerns were raised regarding random selection of participants into intervention and waitlist control conditions in a such a resource-uncertain and time-constrained situation. In response, we chose to initially implement an experimental trial, with non-randomized controls, and only moved to a fully randomized trial once longer-term funding was guaranteed to Mercy Corps for program implementation. At all stages, we relied on Syrian/Jordanian communities and research/humanitarian staff to help us design an ethical, robust, and culturally-compelling means of randomization.

#### Methods

A second set of challenges was related to methods. We began the study with a toolkit comprising psychometric and socio-demographic questionnaires, stress biomarkers, and cognitive tests – then made adjustments in response to pilot studies and fieldwork experience. We wanted to ensure that youth, local communities, and humanitarian staff were fully on board with the science of objective measurement, without placing undue burden for the young people who volunteered their participation in conditions of poverty, intense heat, and unreliable electricity. We were implementing, at three time-points, in four different cities, a fairly long and demanding study protocol, featuring multiple psychometric instruments to include internationally- and regionally-validated scales, relevant to war-affected youth. We augmented the survey with a culturally-grounded measure of resilience and dropped measures that proved less relevant or too burdensome for participants and fieldworkers (such as salivary cortisol). Collecting biological samples (blood spots, cheek swabs, hair cortisol) and testing cognitive skills (RACER tests of working memory and inhibitory control) in difficult field conditions was demanding – but worthwhile to thoroughly assess logistical feasibility and scientific value in humanitarian settings.

#### Dissemination

We also faced challenges in reconciling the different demands and time-frames for dissemination of results. As scholars with multiple teaching, research, and administration responsibilities, we took 18 months to fully analyse all field and lab data. Senior researchers met regularly in person or via skype, discussing and interpreting data to articulate meaningful results, relevant insights, and succinct conclusions. Local research team members were also in continuous dialogue with senior researchers, locally and internationally. In this way, we crystallised the work of an interdisciplinary research consortium through publishing multiple, peer-reviewed academic papers. However, Mercy Corps needed a bottom line regarding program effectiveness, without delay, to influence *No Lost Generation* funding and policy in timely fashion. We sought to accommodate such needs with preliminary reports, but given that rigorous conclusions could not be drawn until the completion of all analyses and the peer-review of scientific evidence, we slowed down the ability of Mercy Corps to share results publically, at least beyond verbal form, and their global influence on youth-focused humanitarian programming. Interdisciplinary work takes time, but can eventually engage different sectors in policy circles, educational sectors, and the media; most recently, an independent filmmaker released a 35-min documentary film of Syrian refugees and the science of resilience, based on our research work [21].

### Research strategies

In response to the above challenges, our research strategies featured many elements to secure engagement and partnership (Table 3).

**Table 3:** Research strategies for engagement and partnership

*Local ownership:* Developing a strong sense of local ownership, through engagement with communities, before implementing the randomized controlled trial; relying on local partners to recruit and track participants; triangulating data on refugee populations.	
*Engaging youth:* Energizing young people with respect to cutting-edge science, and offering professional haircuts to facilitate hair cortisol sample collection; developing and validating an Arabic-language scale to measure resilience, in response to young people’s requests; eliminating the most demanding aspects of the study at T3, such as tests of cognitive function.	
*Sharing findings*: Local and international research partners shared results and dialogue within humanitarian and academic networks - internationally, regionally, and locally.	

#### Local ownership

Through engagement with local partners and communities, we fostered a strong sense of local ownership. Issues of research design and study implementation were clearly open for discussion with research partners and communities. Local staff remarked that team de-briefings were both unusual and refreshing: they could voice concerns, influence scope and delivery, offer viewpoints and solutions. For example, the ethical issue of randomization was debated in back-and-forth conversations with Syrian and Jordanian families, who themselves provided a fair and transparent solution to secure equi-probability of assignment: after completing baseline assessments, each participant would draw one lollipop from an opaque bag, and a coin flip would randomize two lollipop colours to intervention and control groups. Community engagement was also key to avoid sampling biases: while only 14 families declined study participation, we faced significant sample attrition (*n* = 817 at baseline and *n* = 302 after 11 months) due to defunct phone contact numbers and youth who travelled, fell ill, got married, or were busy working. At T3, we offered transport compensation to families who had moved away in order to remove barriers to participation.

#### Engaging youth

Young people were keen to be included in a project that featured stress biomarkers, as this was cutting-edge science; in particular, Syrian refugees welcomed an opportunity to leverage science as a means of telling their story [22]. We also made participation attractive, with well-appreciated measures: we offered toiletries such as combs and toothbrushes as compensation for young people’s participation time, and we hired local male and female hairdressers to style young people’s hair during cortisol sampling. We chose, also, to discontinue experimental testing of cognitive skills at T3, in light of difficulties experienced by youth due to the long duration of testing. Finally, we adapted and validated the Child Youth Resilience Measure (CYRM, 12-items) in response to an explicit request from young people, who asked why assessment was largely focused on traumatic exposures and posttraumatic stress, and did not include measures of strength and hope [12].

#### Sharing findings

Our ultimate goal was the uptake of rigorous research to improve health-related programming in humanitarian settings. To this end, we presented at many events designed to bring together scholars, humanitarian practitioners, and policy-makers, continuing strong engagement with in-country partners well past the project funding dates. This process informed programmatic decisions: given dual evidence of psychosocial and physiological benefits, Mercy Corps integrated elements of the *Advancing Adolescents* stress-attunement approach into its regional livelihood interventions, and - given null impacts for resilience – turned their attention to engaging whole families, rather than individual youth, into resilience-building efforts. Sharing results with Syrian and Jordanian communities also created palpable excitement about the achievements of science in helping people understand what can be done, practically, to address profound stress in vulnerable populations. While some humanitarian and local staff had been sceptical about the need for randomization and the value of including biological markers, young people themselves – once these concepts were explained - welcomed this type of scientific enquiry; they valued playing a role in advancing science, especially with respect to measuring stress (in hair samples) to assess biological impacts.

We found that biological data collection worked well and provided sharp results: specifically, high cortisol levels revealed a body in a state of high alert; chronically low levels indicated blunted sensitivity to environmental challenges; and mixed profiles showed dysregulation [13, 14]. In contexts of war and forced displacement, this has value for understanding how the alleviation of profound stress may protect adolescent health and developmental trajectories. Because we leveraged stress physiology into our research design, evidence from this study has been promoted as proof-of-concept within the fields of humanitarian practice and global mental health. And because we used RACER tests to experimentally assess executive function, we were able to contribute to debates on the relative impacts of trauma exposure versus socio-economic deprivation on youth’s cognitive function [16].

Finally, the project created several professional development opportunities. For example, a Syrian researcher was able to travel to the United States to visit laboratories used for cortisol analysis. Both Syrian/Jordanian and Western senior scientists went to global dissemination meetings and academic conferences, prompting the funders of the study to commend our North-South collaboration as a model partnership. The project prompted new collaborations, including a study on the genetics of stress and resilience [23, 24], research on how an educational intervention for war-affected youth develops, and research on the intergenerational transmission of trauma (forthcoming). Finally, the work directly informed evaluation strategies for organisations such as We Love Reading and Noor Hussein Foundation, in addition to Mercy Corps.

## Conclusions

Reflection on the process of implementing this interdisciplinary evaluation study established four important lessons. First, we learnt that high-quality scientific research is feasible, and can be useful and ethical, in humanitarian settings: with the support of a local research team and active engagement with their community, we were able to realize an ambitious research program to assess the bio-psychosocial and cognitive signatures of profound stress alleviation. Second, we found young people in the context of a humanitarian crisis to be engaged, motivated, and informed regarding the value of scientific research seeking to determine effective means of supporting them in dealing with the profound stress of conflict and displacement.

Third, we learnt that sustained partnerships between scholars, humanitarians, funders, and beneficiaries across multiple locales produces a strong sense of research ownership – back-and-forth discussions of complex challenges and heterogeneous findings help grow the research agenda. Finally, we learnt that opportunities for frequent, meaningful conversations are strongly needed – and that serious dedication to one’s work does not preclude dialogue and humour. Working with multiple, diverse partners often entails bridging different needs and priorities: local communities want their concerns met and recognized; humanitarian organizations are focused on delivering care, and need research findings to support that effort; scholars task themselves with producing research that informs this engagement and meets a high standard for academic scrutiny. Positioned in this mix are the funding agencies disbursing limited resources, the state confronting a challenging crisis, and the media in pursuit of newsworthy stories. Sound partnerships between these multiple actors requires time, funding, good communication, reflexive evaluation, and an ethos of building trust, respect, and meaningful engagement.

## Data Availability

The English and Arabic versions of the Child Youth Resilience Measure (CYRM-12) are published as an online appendix file in Panter-Brick et al. (2018). All data mentioned in this paper are archived in Panter-Brick et al. (2020); the supplementary genetic data are found in Clukay et al. (2019a,b). Clukay, C. Dajani, R., Hadfield, K., Quinlan, J., Panter-Brick, C., & Mulligan, C. (2019a). Association of MAOA genetic variants and resilience with psychosocial stress: A longitudinal study of Syrian refugees. Retrieved from: https://journals.plos.org/plosone/article?id=10.1371/journal.pone.0219385. DOI: 10.1371/journal.pone.0219385 Clukay, C. Matarazzo, A., Dajani, R., Hadfield, K., Panter-Brick, C., & Mulligan, C. (2019b). FAAH, SLC6A4, and BDNF variants are not associated with psychosocial stress and mental health outcomes in a population of Syrian refugee youth [data file and codebook]. Retrieved from: https://data.mendeley.com/datasets/2wbptg7vyn/3. DOI: 10.17632/2wbptg7vyn.3 Panter-Brick, C., Hadfield, K., Dajani, R., Eggerman, M., Ager, A., & Ungar (2018). Resilience in context: A brief and culturally grounded measure for Syrian refugee and Jordanian host-community adolescents. *Child Development, 89*, 1803–1820. Panter-Brick, C., Eggerman, M., Hadfield, K., & Dajani, R., Ager, A. (2020). Beyond self-reports: Testing psychosocial, biological, and cognitive signatures of profound stress in a randomized controlled trial impact evaluation for adolescents affected by the Syria crisis. [data file and codebook]. Retrieved from: https://data.mendeley.com/datasets/x6n4hhk45v/1. DOI: 10.17632/x6n4hhk45v.1

## References

[1] Ager A, Burnham G, Checchi F, Gayer M, Grais RF, Henkens M (2014). Strengthening the evidence base for health programming in humanitarian crises. Science.

[2] Tol WA, Patel V, Tomlinson M, Baingana F, Galappatti A, Panter-Brick C (2011). Research priorities for mental health and psychosocial support in humanitarian settings. PLoS Med.

[3] UNHCR. Global forced displacement tops 70 million. https://www.unhcr.org/news/stories/2019/6/5d08b6614/global-forced-displacement-tops-70-million.html. Accessed 23 Oct 2019.

[4] UNHCR. Operational Portal – Refugee Situations. Syria Regional Refugee Response. https://data2.unhcr.org/en/situations/syria Accessed 19 May 2020.

[5] UNHCR (2020). Syrian regional refugee response in Jordan.

[6] Verma S, Petersen AC (2018). Developmental science and pathways to sustainable development for children and youth. Developmental science and sustainable development goals for children and youth.

[7] Save the Children (2017). Invisible wounds: the impact of six years of war on the mental health of Syria's children.

[8] https://www.nolostgeneration.org/. Accessed 11 Feb 2020.

[9] Mercy Corps. Advancing adolescents approach; 2016. https://www.mercycorps.org/sites/default/files/Mercy_Corps_Advancing_Adolescents_Approach_Lebanon_Jordan_Syrian-10-2016.pdf. Accessed 3 Nov 2019.

[10] Macphail J, Niconchuk M, El-wer N. Conflict, the brain, and community: a neurobiology-informed approach to resilience and community development. In: Phillips R, Kenny S, McGrath B, editors. London: The Routledge handbook of community development, human rights and resilience; 2017. p. 340–57.

[11] Panter-Brick C, Dajani R, Eggerman M, Hermosilla S, Sancilio A, Ager A (2018). Insecurity, distress and mental health: experimental and randomized controlled trials of a psychosocial intervention for youth affected by the Syrian crisis. J Child Psychol Psychiatry.

[12] Panter-Brick C, Hadfield K, Dajani R, Eggerman M, Ager A, Ungar M (2018). Resilience in context: a brief and culturally grounded measure for Syrian refugee and Jordanian host-community adolescents. Child Dev.

[13] Dajani R, Hadfield K, van Uum S, Greff M, Panter-Brick C (2018). Hair cortisol concentrations in war-affected adolescents: a prospective intervention trial. Psychoneuroendocrinology.

[14] Panter-Brick C, Wiley K, Sancilio A, Dajani R, Hadfield K. C-reactive protein, Epstein-Barr virus, and cortisol trajectories in war-affected adolescents: links with stress, mental health, and cognitive function during a randomized controlled trial. Brain Behav Immun. 2019. 10.1016/j.bbi.2019.02.015. 10.1016/j.bbi.2019.02.015 PMC7327518 30797045

[15] Ford CB, Kim HY, Brown L, Aber JL, Sheridan MA (2019). A cognitive assessment tool designed for data collection in the field in low-and middle-income countries. Res Comp Int Educ.

[16] Chen A, Panter-Brick C, Hadfield K, Dajani R, Hamoudi A, Sheridan M (1856). Minds under siege: cognitive functioning in Syrian refugee adolescents impacted by armed conflict and displacement. Child Dev.

[17] Metzler J, Savage K, Yamano M, Ager A. Evaluation of child friendly spaces: an inter-agency series of impact evaluations in humanitarian emergencies: Columbia University Mailman School of Public Health & World Vision International; 2015. https://www.wvi.org/sites/default/files/Evaluation%20of%20CFS_Final%20Research%20Report_0.pdf Accessed 5 Nov 2019.

[18] Bangpan M, Dickson K, Felix L, Chiumento A (2017). The impact of mental health and psychosocial support interventions on people affected by humanitarian emergencies: a systematic review humanitarian evidence programme.

[19] Hermosilla S, Metzler J, Savage K, Musa M, Ager A (2019). Child friendly spaces impact across five humanitarian settings: a meta-analysis. BMC Public Health.

[20] Panter-Brick C, Kurtz J, Dajani R (2018). What strong partnerships achieve: innovations in research and practice. Humanitarian Exchange.

[21] Bourke R (2020). Terror and Hope: the science of resilience.

[22] Underwood E. Lessons in resilience: in war zones and refugee camps, researchers are putting resilience interventions to the test. Science. 2018:976–9. 10.1126/science.359.6379.976 29496858

[23] Clukay CJ, Dajani R, Hadfield K, Quinlan J, Panter-Brick C, Mulligan C (2019). Association of MAOA genetic variants and resilience with psychosocial stress: a longitudinal study of Syrian refugees.

[24] Clukay CJ, Matarazzo A, Dajani R, Hadfield K, Panter-Brick C, Mulligan CJ. FAAH, SLC6A4, and BDNF variants are not associated with psychosocial stress and mental health outcomes in a population of Syrian refugee youth. BioRxiv. 2019;1–16.

